# Modality-AGnostic Image Cascade (MAGIC) for Multi-Modality Cardiac Substructure Segmentation

**Published:** 2025-06-12

**Authors:** Nicholas Summerfield, Qisheng He, Alex Kuo, Ahmed I. Ghanem, Simeng Zhu, Chase Ruff, Joshua Pan, Anudeep Kumar, Prashant Nagpal, Jiwei Zhao, Ming Dong, Carri K. Glide-Hurst

**Affiliations:** 1. Department of Medical Physics, University of Wisconsin-Madison, Madison, Wisconsin; 2. Department of Human Oncology, University of Wisconsin-Madison, Madison, Wisconsin; 3. Department of Computer Science, Wayne State University, Detroit, Michigan; 4. Department of Radiation Oncology, Henry Ford Cancer Institute, Detroit, Michigan; 5. Alexandria Department of Clinical Oncology, Faculty of Medicine, Alexandria University, Alexandria, Egypt; 6. Department of Radiation Oncology, The Ohio State University, Columbus, Ohio; 7. Department of Radiology, University of Wisconsin-Madison, Madison, Wisconsin; 8. Department of Biostatistics and Medical Informatics, University of Wisconsin-Madison, Madison, Wisconsin

## Abstract

**Purpose:**

Cardiac substructures are essential in thoracic radiation therapy planning to minimize risk of radiation-induced heart disease. Deep learning (DL) offers efficient methods to reduce contouring burden but lacks generalizability across different modalities and overlapping structures. This work introduces and validates a Modality-AGnostic Image Cascade (MAGIC) for comprehensive and multi-modal cardiac substructure segmentation.

**Materials and methods:**

MAGIC is implemented through replicated encoding and decoding branches of an nnU-Net-based, U-shaped backbone conserving the function of a single model. Twenty cardiac substructures (heart, chambers, great vessels (GVs), valves, coronary arteries (CAs), and conduction nodes) from simulation CT (Sim-CT), low-field MR-Linac, and cardiac CT angiography (CCTA) modalities were manually delineated and used to train (n=76), validate (n=15), and test (n=30) MAGIC. Twelve comparison models (four segmentation subgroups across three modalities) were equivalently trained. All methods were compared for training efficiency and against reference contours using the Dice Similarity Coefficient (DSC) and two-tailed Wilcoxon Signed-Rank test (threshold, p<0.05).

**Results:**

Average DSC scores were 0.75±0.16 (Heart, 0.96±0.01; Chambers, 0.89±0.05; GVs, 0.81±0.09; CAs, 0.60±0.13; Valves, 0.70±0.18; Nodes, 0.66±0.12) for Sim-CT, 0.68±0.21 (Heart, 0.94±0.01; Chambers, 0.87±0.05; GVs, 0.72±0.18; CAs, 0.50±0.18; Valves, 0.62±0.16; Nodes, 0.52±0.16) for MR-Linac, and 0.80±0.16 (Heart, 0.95±0.01; Chambers, 0.93±0.04; GVs, 0.84±0.06; CAs, 0.77±0.12; Valves, 0.68±0.23; Nodes, 0.72±0.11) for CCTA. MAGIC outperforms the comparison in 57% of cases, with limited statistical differences.

**Conclusions:**

MAGIC offers an effective and accurate segmentation solution that is lightweight and capable of segmenting multiple modalities and overlapping structures in a single model. MAGIC further enables clinical implementation by simplifying the computational requirements and offering unparalleled flexibility for clinical settings.

## Introduction

Radiation-induced heart disease is a known complication of thoracic radiation therapy (RT) encompassing both acute and late effects^1,2^. The current standard of care is to delineate the whole heart as a single organ at risk and use simple metrics to evaluate dose-to-volume relationships for potential cardiac risk^3^. However, radiation dose to cardiac substructures has been more strongly associated with cardiotoxicities^1^ and has been proposed as targets for sparing in advanced cardiac sparing RT plans^4,5^.

Deep learning-based auto-segmentation for cardiac substructures have offered efficient solutions to implement for RT^6^. Yet, at present, these methods are unimodal, targeted to a specific image type such as computed tomography (CT) or magnetic resonance (MR) imaging, limiting inclusion in clinical workflows due to specialized contouring, time demands, and the lack of clinically integrated automatic segmentation techniques. Radiotherapy has historically been predominantly CT-based^6–11^. However, with the rise of MR-guided RT^12^ (MR coupled with linear accelerators, MR-Linac) and the availability of cardiac gated technology (e.g., cardiac CT angiography (CCTA) on CT simulators (Sim-CT)^13,14^), multiple imaging modalities can now be used to evaluate cardiac substructure anatomy. Recent methods target a specific modality and are incapable of generalizing to different inputs without large performance loss of prediction failure^15,16^ and limit the advantages of different contrasts and resolutions available between modalities. Morris et al.^11^ incorporated paired Sim-CT and T2-weighted MR images during training to aid in model fitting and boost accuracy, yet clinical use remained unimodal in application to Sim-CT inputs. To handle the wide array of treatment and imaging modalities available in radiotherapy, in the past, modality-specific, unimodal models have been required.

Cardiac anatomy is complex and generally considered to encompass the whole heart, chambers, great vessels, coronary arteries, valves, and the conduction system. When combined into a composite label, several of these structures overlap one another with more than one true label per image voxel, thereby complicated segmentation tasks. Semantic segmentation is incapable of handling overlapping labels in a single model and methods presented by Momin et al.^7^, Chen et al.^8^, and van der Pol et al.^9^ employ specifically trained models to tackle subgroups of non-overlapping structures. Harms et al.^10^ applied instance segmentation which handles overlap by first extracting structure-specific regions followed by subgroup-specific semantic segmentation layers to form masks. Each subgroup-specific model must be further fine-tuned and optimized to accurately segment a composite mask.

At least four models would be necessary to automatically segment all overlapping cardiac substructures currently of interest in the literature^7–10^. Furthermore, a unique set of models is required to handle different imaging modalities. Each individual model requires a curated and annotated dataset, lengthy optimization, clinical validation, and continued maintenance to ensure sustained performance^17^, hindering scalability when additional datasets become available and widespread clinical adoption. To address these current limitations, we present a Modality AGnostic Image Cascade (MAGIC) framework. MAGIC is unique in that it is a lightweight method for image segmentation that generalizes to all modalities included during optimization while predicting overlapping labels to a single modality input image within a one flexible model. We implement MAGIC on a state-of-the-art deep-learning backbone, targeting 20 cardiac substructures including conduction nodes, and benchmark its performance against standard approaches of uniquely trained, individual modality models, thereby laying the technical groundwork and generalizability for future prospective modality-agnostic clinical trials.

## Methods and Materials

### Patient Cohort

Three imaging modalities were retrospectively reviewed in this institutional review board-approved study from two different institutions: simulation (Sim-) CT (n = 65), 0.35T MR-Linac (n = 34 unique patients, 2–3 fractionated images per patient used as training augmentations^12^), and Cardiac CT Angiography (CCTA) (n = 65). Both MR-Linac and Sim-CT images were obtained from patients undergoing RT at one of two institutions for either thoracic or abdominal cancers adjacent to the heart. Diagnostic CCTA images were collected from a single institution following standard clinical protocols, with a field of view encompassing the heart.

Sim-CT patients were imaged without contrast on either a Brilliance Big Bore CT simulator (Philips Medical Systems, Cleveland, OH) or a Somatom Edge CT scanner (Siemens Healthineers, Malvern, PA). Images were acquired with 0.98–1.4×0.98–1.4×2–3 mm^3^ resolution, using 120–140 kVp, 100–434 mAs, and no added intravenous contrast. Patients were immobilized in the supine position and imaged using either free breathing or averaged or respiratory gated four-dimensional CT scans. CCTA patients were imaged with 60–100 ml of intravenous contrast, 80–140 kVp, and 1 mAs following clinical protocols. CCTA images were reconstructed at a resolution of 0.49×0.49×0.63mm^3^ with a vendor-provided deep learning algorithm (SnapShot Freeze 2, GE Healthcare) to minimize cardiac motion. MR-Linac patients were imaged on a 0.35T MR-linac (ViewRay Systems, Denver, CO) at both institutions using clinically available T1/T2 balanced steady state free precession sequences (TrueFISP, Siemens, MAGNETOM Avanto, Syngo MR B19) with the following parameters: scan time=17–25 seconds, resolution=1.5–1.6×1.5–1.6×3 mm^3^, flip angle=60°, and phase encoding in anterior-poster plane.

All images were manually labeled with 20 cardiac substructures including whole heart (WH); chambers (left/right atrium/ventricle (LA, RA, LV, RV)); great vessels (ascending aorta (AA), superior/inferior vena cava (SVC, IVC), and pulmonary artery/veins (PA, PVs)); coronary arteries (right (RCA), left main (LMCA) left anterior descending (LADA), and left circumflex (CLFX) arteries); valves (aortic (V-AV), pulmonic (V-PV), mitral (V-MV), and tricuspid (V-TV) valves); and conduction nodes (sinoatrial (N-SA) and atrioventricular (N-AV) nodes). All 20 substructures were manually contoured following a consensus of published guidelines^18–21^ and expert consultation with a radiologist with cardiovascular subspeciality and 10+ years of experience.

### MAGIC Methods

**Figure 1:**
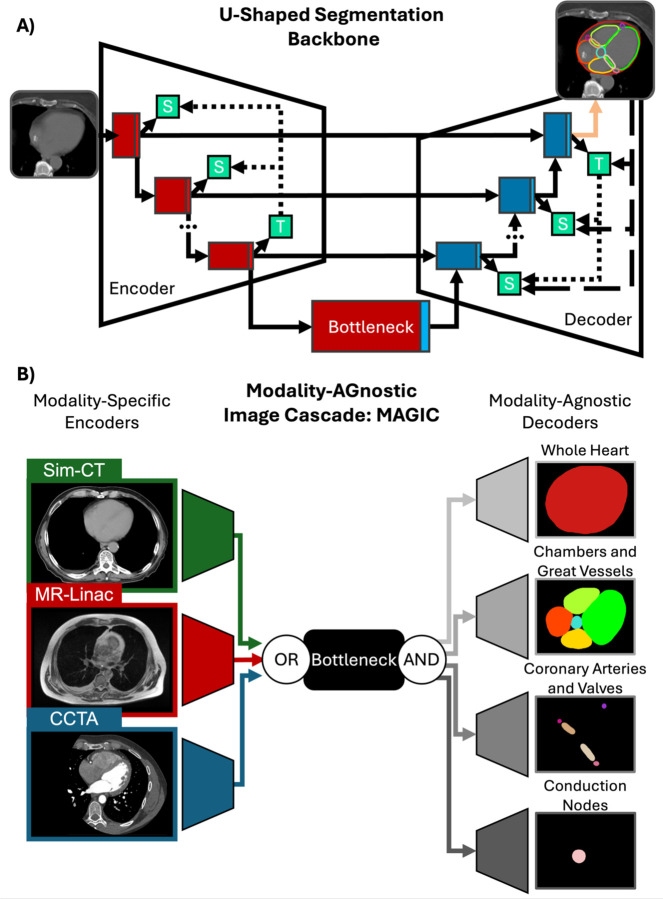
Example U-shaped segmentation backbone (A, top) and Modality-AGnostic Image Cascade (MAGIC, B, Bottom). The modality-agnostic and multi-label capabilities of MAGIC arise from duplicated modality-specific encoders and modality-agnostic decoders connected to a single bottleneck. Backbone characteristics such as skip connections and deep supervision are preserved within the framework.

To facilitate multi-modal semantic segmentation of overlapping structures, we developed Modality-AGnostic Image Cascade (MAGIC), as shown in Figure 1B. Modality-agnostic segmentation is accomplished by utilizing modality-specific encoders^15,16^, whereas overlapping structures are handled through multiple decoder branches, each targeting a group of non-overlapping structures. All branches are connected to a shared bottleneck, resulting in a single unified model. By optimizing the shared bottleneck and decoder branches with all modalities, cross-modality learning enables the model to share information between inputs, which has been shown to improve model robustness^15,16^. Similarly, by optimizing all semantic segmentation groups with the same encoding branches, cross-correlation between structures is preserved, further improving prediction confidence^22^. During model fitting, a single optimizer is attached to all branches to harmonize learning and preserve gradient momentum across modalities^23^. At each iteration, an image is randomly selected from all available datasets to avoid biasing the model towards any single modality.

MAGIC is a framework that can be applied to any U-shaped network as shown in Figure 1A, by replicating encoding and decoding branches to match the dataset. Optimization and architectural designs—including skip connections, deep supervision, and self-distillation—remain applicable across the MAGIC framework by mimicking the operations of a single model. Building upon our prior work, we implemented MAGIC with a modified nnU-Net with dual self-distillation (nnU-Net.wSD) that was validated for cardiac segmentation^12,24^. Three encoder branches for MR-Linac, Sim-CT, and CCTA images, and four decoder branches were initialized for the following subgroups of cardiac substructures: A) whole heart; B) chambers and great vessels; C) coronary arteries and valves; D) conduction nodes. To compare our single MAGIC to conventional approaches, twelve “unimodal” models (four segmentation subgroups across three modalities) were trained^7–10^.

### Training Methods

All work was conducted in Python (version 3.10) using the open-source packages PyTorch^25^ (version 2.1) and MONAI^26^ (version 0.9), with training and evaluation performed on an in-house Nvidia A4000 graphics processing unit with 48 GB of memory. MAGIC was trained twice, utilizing both fully- and semi-supervised optimization to further expand the dataset and improve model robustness^27^. First, manually labeled datasets for each modality were split for testing (n=10), validation (n=5), and training (Sim-CT and CCTA, n=25; MR-Linac, n=26) an initial model that was used to predict on unlabeled images. Following visual assessment, an additional 25 images per modality were included in the training sets to reoptimize MAGIC. The same datasets of manually labeled and predicted images were used to equivalently train all unimodal models.

To improve generalizability^28,29^, all images underwent similar pre-processing steps and training augmentations. CT-based images were windowed to [−250, 550] HU and all modalities underwent k-score normalization. Images were resampled to 1.5×1.5×1.5 mm^3^ resolution and semi-automatically cropped to the whole heart with manual verification. Training augmentations were applied to each image prior to input during fitting, including random flipping along each axis; rotations of both 90° and between −10° and 10° along each axis; shifting and scaling pixel intensity between −10% and 10%; and randomly cropping the images to smaller patches of 96×96×96 voxels.

Optimization was constrained through minimizing a hybrid loss function. Segmentation is primarily guided through a combined Dice, Dice-boundary, and cross-entropy loss function with equal weighting. The Dice loss addresses the label imbalance inherent to segmentation tasks, the Dice-boundary loss applies emphasis on the surface shape of the structure, and the cross-entropy loss aids in label classification^30^. The nnU-Net.wSD backbone of MAGIC and unimodal models utilizes additional losses to facilitate dual-self distillation during optimization^24^ which are combined linearly with equal weighting to the primary loss. Models were optimized for 500 epochs using an AdamW optimizer (learning rate, 10^−3^; weight decay, 10^−5^) and poly learning rate reduction (gamma, 0.9). For full-volume predictions during validation and testing, sliding-window inference is used with an overlap of 90% to retain flexibility of input shape. The hold-out validation set was evaluated after each epoch to identify the best model configuration based on the Dice similarity coefficient (DSC) averaged across all substructures and modalities.

### Evaluation techniques

All test predictions from the singularly trained MAGIC and 12 unimodal models (four segmentation subgroups across three modalities) were compared against the reference contours. Model efficiency was evaluated by comparing the time requirements for training and inference, training memory requirements, and quantity of trainable parameters between methods. Overall performance was measured quantitatively through DSC, surface DSC (sDice; tolerance, 2 voxels), 95% Hausdorff Distance (HD95), and mean surface distance (MSD) ^31^. Due to their tubular nature, the coronary arteries were further evaluated using a novel metric, centerline Dice^32^, as applied to brain and retina vessel segmentation, and centerline visualization. All quantitative metrics comparing MAGIC and unimodal models were assessed through two-tailed Wilcoxon signed-rank tests with p<0.05 considered significantly different. In case a model fails to predict a structure, the HD95 and MSD datapoint is treated as infinite and discarded, using a two-tailed Mann-Whitney U statistical test for unpaired data to assess statistical differences (threshold of p<0.05). Finally, Qualitative visualization of predictions was performed, showing the predicted against reference contours overlaid on the underlying anatomy.

## Results

All models were trained independently on a graphics processing unit. The baseline Unimodal models for each imaging modality required a total of 185 hours, maximally 14 GB (43 GB combined) of memory, and 736 million parameters to train. In contrast, MAGIC required only 31 hours (an 83% reduction), 32 GB of memory, and 194 million parameters (a 74% reduction) to train. When used during inference, the unimodal models required 15.3 seconds to generate a composite label whereas MAGIC required 11.4 seconds.

**Figure 2:**
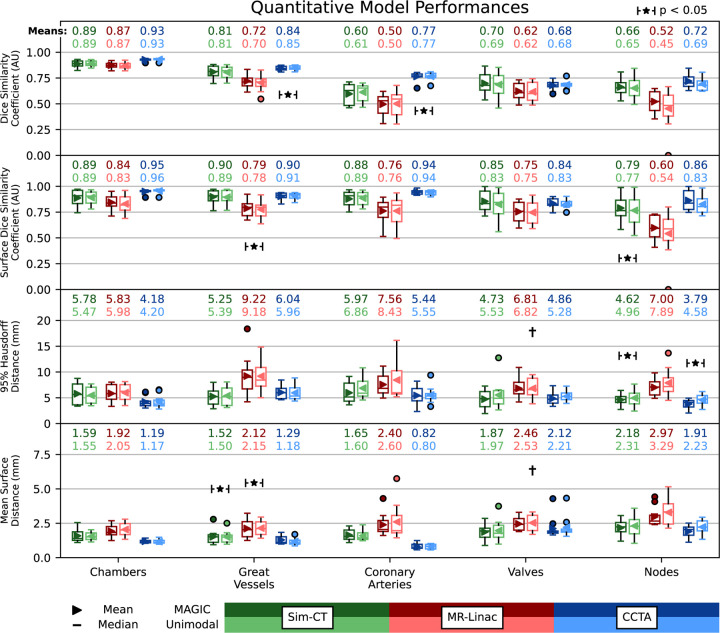
Quantitative model performance comparing MAGIC against 12 specialized comparator models examining the Dice Similarity Coefficient (DSC, AU), Surface Dice (AU, tolerance, 2 voxels), 95% Hausdorff Distance (HD95, mm), and Mean Surface Distance (MSD, mm) for Sim-CT (top), MR-Linac (middle), and CCTA (bottom) inputs. † comparator model failed to predict the atrioventricular node on a test patient resulting in an infinite metric for HD95 and MSD and was discarded for comparisons.

Quantitative results are shown in Figure 2, comparing the singularly trained MAGIC model against unimodal models for substructure group averages per modality. Whole heart MAGIC predictions were above 0.94 DSC and not included in the figure. MAGIC conserves strong overall agreement with reference contours across all three modalities. Composite DSC scores across all 20 structures were 0.75±0.16 (sDice, 0.87±0.13; HD95, 5.3±2.8mm; MSD, 1.7±0.8mm) for Sim-CT, 0.68±0.21 (sDice, 0.77±0.18; HD95, 7.3±4.8mm; MSD, 2.3±1.2mm) for MR-Linac, and 0.80±0.16 (sDice, 0.90±0.14; HD95, 5.1±3.6mm; MSD, 1.4±1.2mm) for CCTA images. The unimodal model for MR-Linac cardiac node segmentation failed to predict N-AV for one of the test cases whereas MAGIC made a prediction. MAGIC outperformed for 42, 58, and 37 of 80 metric comparisons for Sim-CT, MR-Linac, and CCTA respectively. Of the underperformances, MAGIC was within 0.02 for Dice and sDice or 1mm for HD95 and MSD for 35 of 38, 20 of 22, and 41 of 43 comparisons. Overall, MAGIC’s performance was comparable to that of 12 unimodal models (four segmentation subgroups across three modalities) with limited statistical differences across comparisons.

**Figure 3:**
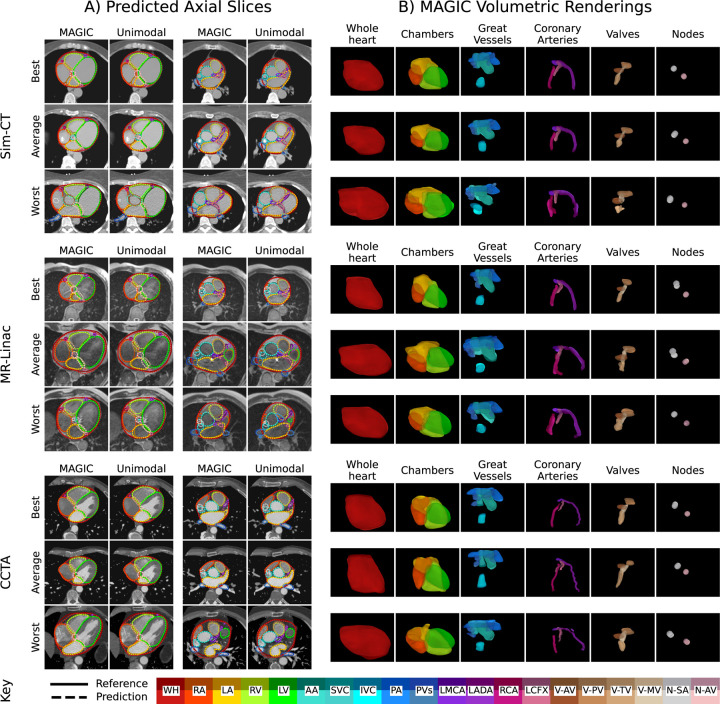
Qualitative analysis highlighting best, average, and worst results chosen by patient-average Dice similarity coefficient across Sim-CT (top), MR-Linac (middle), and CCTA (bottom) inputs. A) Axial slices comparing MAGIC and unimodal predictions against reference contours. B) Volumetric renderings of MAGIC predictions overlayed with reference contours. Abbreviations: Ref, Reference; Pred, Prediction; WH, whole heart; RA, Right Atrium; LA, Left Atrium, RV, Right Ventricle; LV, Left Ventricle; AA, Ascending Aorta; SVC, Superior Vena Cava; IVC, Inferior Vena Cava; PA, Pulmonary Artery; PV, Pulmonary Veins; LMCA, left main coronary artery; LADA, Left Anterior Descending Artery; RCA, Right Coronary Artery; LCFX, Left Circumflex Artery; V-AV, Aortic Valve; V-PV, Pulmonic Valve; V-TV, Tricuspid Valve; V-MV, Mitral Valve; N-SA, Sinoatrial Node; N-AV, Atrioventricular Node.

Qualitative axial comparisons for 4-chamber and basal views and volumetric renderings are shown in Figure 3. Best, average, and worst representations were chosen off patient-average DSC. The best cases demonstrate strong visual consistency with the reference contours, with the primary discrepancies observed at the superior/inferior extents of the predicted structures. The worst Sim-CT is of a 50% gated 4D CT image with visually lower image quality compared to the best and average cases, contributing to reduced overall performance. The average and worse MR-Linac cases were impacted by artifacts where both models were unaffected in the 4-chamber view (Figure 2, Middle, Lower); however, MAGIC shows a discontinuity in the LCFX in the basal view (Figure 2, Middle, Middle). Finally, all CCTA cases show strong agreement, with the primary areas of disagreement arising from conflicting coronary veins or sinus that visually resemble coronary arteries in some scans.

**Figure 4:**
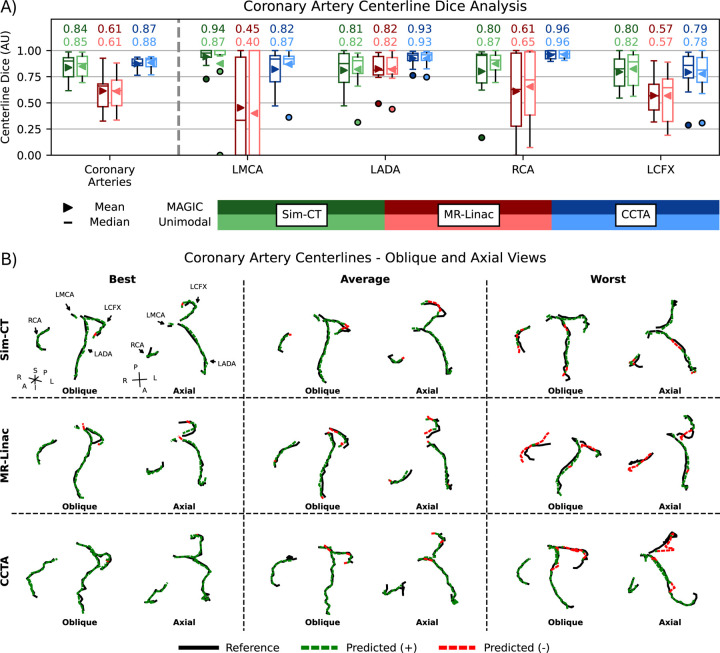
Centerline Dice performance comparing MAGIC and unimodal methods (top) and qualitative centerline review of MAGIC predictions against reference contours (bottom). Best, average, and worst predictions based off centerline Dice. A positive (+, green) prediction corresponds with the centerline remaining within the reference contour and negative (-, red) predictions deviate outside. Abbreviations: Left Main Coronary Artery, LMCA; Left Anterior Descending Artery, LADA; Right Coronary Artery, RCA; Left Circumflex Artery, LCFX.

Coronary artery analysis is presented in Figure 4 for centerline Dice metrics across all three modalities (Figure 4A) and visual comparisons of MAGIC predicted centerlines against reference contours (Figure 4B). No significant differences were found when comparing methods. For each modality, the best-case predictions achieved centerline Dice scores greater than 0.93 with centerlines that fall nearly completely within the reference contours. Predicted coronary arteries closely match both the tract and length of the reference in the average cases. Greater deviations in posterior-inferior regions were present in the worst cases for some RCA and LCFX predictions whereas LADA predictions consistently have centerline Dice scores >80%, showing high agreement even in the worst cases.

## Discussion

This work introduced and validated MAGIC, a lightweight DL framework applicable to multiple imaging modalities and capable of segmenting overlapping cardiac substructures in a single model. By incorporating multiple modality-specific encoders coupled with a shared modality-agnostic bottleneck and decoder branches, MAGIC offers a flexible segmentation solution for universal segmentation. MAGIC performs comparably to unimodal counterparts yet offers a substantially simplified approach, achieving >80% reduction in training time, faster inference speeds, and requiring >70% fewer parameters. The framework is compatible with any U-shaped semantic segmentation backbone and optimization strategy, as demonstrated here using a start-of-the-art nnU-Net backbone with dual-self distillation^12,24^.

On Sim-CT inputs, MAGIC scored an average DSC of 0.75±0.16 (Heart, 0.96±0.01; chambers, 0.89±0.05; great vessels, 0.81±0.09; coronary arteries, 0.60±0.13; valves, 0.70±0.18; nodes, 0.66±0.12) and HD95 of 5.3±2.8mm (heart, 4.3±1.5mm; chambers, 5.8±2.9mm; great vessels, 5.3±2.8mm; coronary arteries, 6.0±3.2mm; valves, 4.7±2.6mm; nodes, 4.6±1.7mm). Recently, Chen et al.^8^ proposed four nnU-Nets to segment 19 cardiac substructures from Sim-CT images targeted to the whole heart (DSC, 0.95; HD95, 4.1mm), chambers (DSC, 0.91; HD95, 4.4mm), great vessels (DSC, 0.86; HD95 6.0mm), valves (DSC, 0.81; HD95, 4.4mm), and coronary arteries (DSC, 0.60; HD95, 7.3mm). Our single MAGIC performed comparably for all structures except for the valves. MAGIC holds comparable spatial accuracy (composite HD95 <6mm) but lower DSC, potentially due to different in-plane spatial resolution (<1mm compared to 1.5mm) and a larger modality-specific training dataset (n=75 vs n=25 manually delineated Sim-CT). Van der Pol et al.^9^ similarly proposed two nnU-Nets to segment 16 cardiac substructure from Sim-CT images, including chambers (DSC, ~0.89; HD95, ~6.5mm), great vessels (DSC, ~0.78; HD95, ~11.1mm), valves (DSC, ~0.35; HD95, ~11.60mm), and coronary arteries (DSC, ~0.35; HD95, ~11.6mm). MAGIC is comparably offering superior DSC and HD95 on these structures with similar Sim-CT imaging (resolution 0.87–1.56×0.87–1.56×1–3 mm^3^). However, performance difference likely arise in patient populations as ventricular tachycardia patients may have increased imaging artifacts due to surgical implants compared to typical Sim-CT datasets.

Cardiac segmentation has recently been extended to include low-field MR-Linac images^12^. In this study, a single nnU-Net with dual self-distillation was applied to segment 12 non-overlapping structures including chambers (DSC, 0.85; HD95, 7.0mm), great vessels (DSC, 0.67; HD95, 9.9mm), and coronary arteries (DSC, 0.33; HD95, 9.5mm). MAGIC offered improved performance for the chambers (DSC, 0.87; HD95, 5.8mm), great vessels (DSC, 0.72; HD95, 9.2mm), and coronary arteries (DSC, 0.50; HD95, 7.6mm) with added contours for the whole heart, valves, and conduction nodes. Between the presented modalities, MAGIC had the lowest performance on MR-Linac data. This is potentially due to low-field MR-Linac images having lower image resolution compared to CT (MR-Linac, 1.5–1.6×1.5–1.6×3mm^3^; Sim-CT, 0.98–1.4×0.98–1.4×2–3 mm^3^) and more prone to artifacts^12^. MAGIC performance may be improved by complimenting the low-field dataset with the same sequences from a high-field MR-Linac^4^.

Diagnostic CCTA has been utilized for cardiac segmentation, but limited comparisons exist for composite labels of multiple structures. Zhuang et al.^33^ reported model performances for the Multi-Modality Whole Heart Segmentation grand challenge for CCTA targeted to chambers and great vessels wherein the best method has a combined DSC of 0.91 and Hausdorff Distance (HD) of 25.2mm. On comparable structures, MAGIC has an equivalent DSC of 0.91 but far superior HD of 9.5mm (HD95, 4.9mm). CCTA is commonly used for coronary artery segmentation but often as a single binary task where all coronary arteries are considered as a single classification. Gharleghi et al.^34^ reported model performance for such a binary coronary artery segmentation grand challenge wherein the best method has a DSC of 0.87 and HD95 of 4.2mm. Comparatively, MAGIC aims to further classify the coronary arteries into four unique structures, achieving an average DSC of 0.77 and HD95 of 5.4mm. Here, the decreased DSC is likely due to MAGIC being targeted to a more complex task with lower image resolution yet spatial precision is conserved with a comparable HD95.

Coronary artery segmentation is difficult on RT imaging as their thin, tube-like geometry is obstructed by motion and lower soft tissue contrasts. This geometry further challenges model evaluation as small deviations in space largely impact volumetric metrics such DSC. To critically evaluate coronary artery performance, we examined the centerlines of these vessels. MAGIC scored average centerline Dice^32^ of 0.84, 0.61, and 0.87 for Sim-CT, MR-Linac, and CCTA respectively, with no statistical deviations from unimodal comparators. MR-Linac scored the lowest with an LMCA average of 0.45 but maintains an HD95 of 6.3mm. This lower performance is likely due to disagreements in the superior-inferior direction in addition to the short length of the LMCA. On Sim-CT and CCTA, structure averages range between 0.79 and 0.96, suggesting strong overall predictions for the coronary arteries. Visual inspection of predicted centerlines demonstrated strong agreement with little to no deviations of predicted centerlines outside of reference contours for most structures. The LCFX had the most uncertainty in the superior extent as seen on the worst cases of both MR-Linac and CCTA cases.

MAGIC extends deep learning methods to include the conduction nodes. The cardiac nodes have recently been proposed as an additional sparing structure due to strong connections with conduction disorders ^21^. Loap et al. ^35^ utilized a multi-atlas auto-segmentation, achieving a median DSC of 0.56 and 0.16 for the N-SA and N-AV respectively. Finnegan et al.^36^ proposed a geometric based approach, achieving an average DSC of 0.47 (HD95, 8.7mm) and 0.46 (HD95, 8.4mm) for the N-SA, and N-AV respectively. In comparison, MAGIC scored a DSC of 0.65 (HD95, 4.81mm) and 0.67 (HD95, 4.43mm) for N-SA and N-AV respectively on Sim-CT. MAGIC also presents promising performance on both MR-Linac (DSC, 0.52; HD95, 7.0mm) and CCTA images (DSC, 0.72; HD95: 3.8mm).

One limitation of this study is that no external validation of MAGIC was performed. Although images were gathered from two institutions and multiple observers contribute to the dataset, no external datasets or observers from outside institutions were used. Additionally, model predictions were quantitatively compared to reference contours but fails to grasp direct clinical utility. This work may be improved by performing a 5-point Likert comparison or dosimetric comparison on RT images^8^.

Another limitation in this work is a narrow scope of included modalities. Sim-CT, MR-Linac, and CCTA images were included when training MAGIC. A natural extension of MAGIC would be appending the modality-specific encoders to include images such as dual-energy and calcium scoring CT, high-field MR-Linac, and diagnostic MR for cardiac segmentation. Furthermore, although MAGIC is flexible to input, images used for radiotherapy and diagnostic purposes have different imaging properties. Whereas Sim-CT and low-field MR-Linac images tend to have resolutions of ~1–1.5mm isotropic in-plane and ~1.5–3mm through-plane, diagnostic CCTA is on the order of ~0.5mm in each dimension. All images used were resampled to that of a consistent 1.5mm isotropic resolution which may negatively impact model performance when targeting CCTA images due to information loss. Instead, MAGIC may be improved by using a more equivalent resolution across all included modalities or by training MAGIC with different input resolutions utilized as an additional training augmentation akin to image scaling^37^.

## Conclusion

In conclusion, we have developed and validated MAGIC, a modality-agnostic deep-learning pipeline, applicable to any U-shaped semantic segmentation backbone, for composite cardiac substructure segmentation. We implemented MAGIC on Sim-CT, MR-Linac, and CCTA images and targeted to 20 substructures including whole heart, chambers, great vessels, coronary arteries, valves, and conduction nodes. MAGIC produces competitive predictions against 12 state-of-the-art unimodal models, representing the standard way of handling four segmentation groups across three modalities. Furthermore, MAGIC reduced required training time by >80% and requires >70% less parameters with ~33% faster inference time. This work further enables future prospective clinical trials by simplifying the computational requirements and offering an unparalleled flexibility to the patient setting.

## Data Availability

Research data are not available at this time.
